# Association between oxidative stress-related IGF-1 and prognosis after ischemic stroke: a systematic review and meta-analysis

**DOI:** 10.3389/fneur.2025.1597114

**Published:** 2026-01-09

**Authors:** Xinyu Liu, Jun Wang, Congcong Wang

**Affiliations:** 1 Department of Endocrinology Jinhua People's Hospital, Jinhua City, Zhejiang Province, China; 2 Department of Orthopedics, Rehabilitation Hospital Affiliated to National Research Center for Rehabilitation Technical Aids, Beijing, China; 3 Weifang Second People's Hospital, Weifang, China; 4 Weifang First People's Hospital, Weifang, China; 5 National Research Center for Rehabilitation Technical Aids, Beijing, China; 6 Department of Joint Surgery, Weifang People's Hospital, Shandong Second Medical University Weifang, Weifang, Shandong, China

**Keywords:** oxidative stress, insulin-like growth factor, acute ischemic stroke, meta-analysis, poorprognosis

## Abstract

**Background and Purpose:**

Insulin-like growth factor-1 (IGF-1), an oxidative stress–related neurotrophic factor, has been investigated in stroke due to its potential roles in neuronal survival and vascular regulation. However, findings on its association with ischemic stroke and functional prognosis remain inconsistent. This review aimed to evaluate the association between circulating IGF-1 levels and (1) the risk of ischemic stroke and (2) post-stroke outcomes.

**Methods:**

We systematically searched EMBASE, MEDLINE, PubMed, Google Scholar, and the Cochrane Library from inception to June 2021, and updated the search to November 2025. Case-control or cohort studies reporting risks or odds ratios with 95% confidence intervals were included. Summary estimates were pooled using random-effects models when heterogeneity was substantial.

**Results:**

Our systematic literature search identified 10 articles. Four of these studies examined the association between IGF-1 and the risk of acute ischemic stroke, while the remaining six studies focused on the relationship between IGF-1 and unfavorable outcomes following acute ischemic stroke. No significant association was found between IGF-1 and ischemic stroke risk [risk ratio (RR) = 1.69, 95% confidence interval (CI) = 0.97–2.92, *I*^2^ = 82.2%, random-effects model], nor was there a significant impact of IGF-1 on unfavorable outcomes after ischemic stroke (RR = 1.55, 95% CI = 0.89–2.68, *I*^2^ = 86.6%, random-effects model). However, in the subgroup analysis of IGF-1's effect on unfavorable outcomes after ischemic stroke, IGF-1 was significantly associated with poor prognosis more than 1 year after stroke onset (RR = 3.33, 95% CI = 2.19–5.05, *I*^2^ = 0%, random-effects model).

**Conclusions:**

This systematic review and meta-analysis suggests that oxidative stress-related IGF-1 may be associated with long-term unfavorable outcomes following ischemic stroke, particularly beyond 1 year after onset. However, no significant associations were found with the incidence of acute ischemic stroke or short-term outcomes. Given the observational nature of the included studies and inconsistencies in adjustment for confounders, these findings should be interpreted cautiously. IGF-1 may serve as a potential prognostic biomarker for long-term functional decline after stroke, but further large-scale prospective studies are needed to clarify causality and clinical applicability.

## 1 Introduction

Stroke is one of the leading causes of mortality and long-term disability worldwide, with ischemic stroke representing the most common subtype (1). Despite advances in acute reperfusion therapy, many stroke survivors continue to experience persistent neurological deficits and limited functional recovery (1). This highlights the importance of investigating biological pathways that contribute to ischemic brain injury and long-term outcome.

Insulin-like growth factor 1 (IGF-1), a peptide hormone widely studied in ischemic brain injury models, has shown significant neuroprotective effects. Experimental studies have demonstrated that delayed IGF-1 administration can reduce hypoxia-ischemia-induced neuronal damage and improve behavioral recovery in immature and adult ischemic stroke models (2–5). In addition to direct neuronal protection, estrogen–IGF-1 interactions play an important role in modulating neuroprotection following focal cerebral ischemia (6, 7).

**Figure 1:**
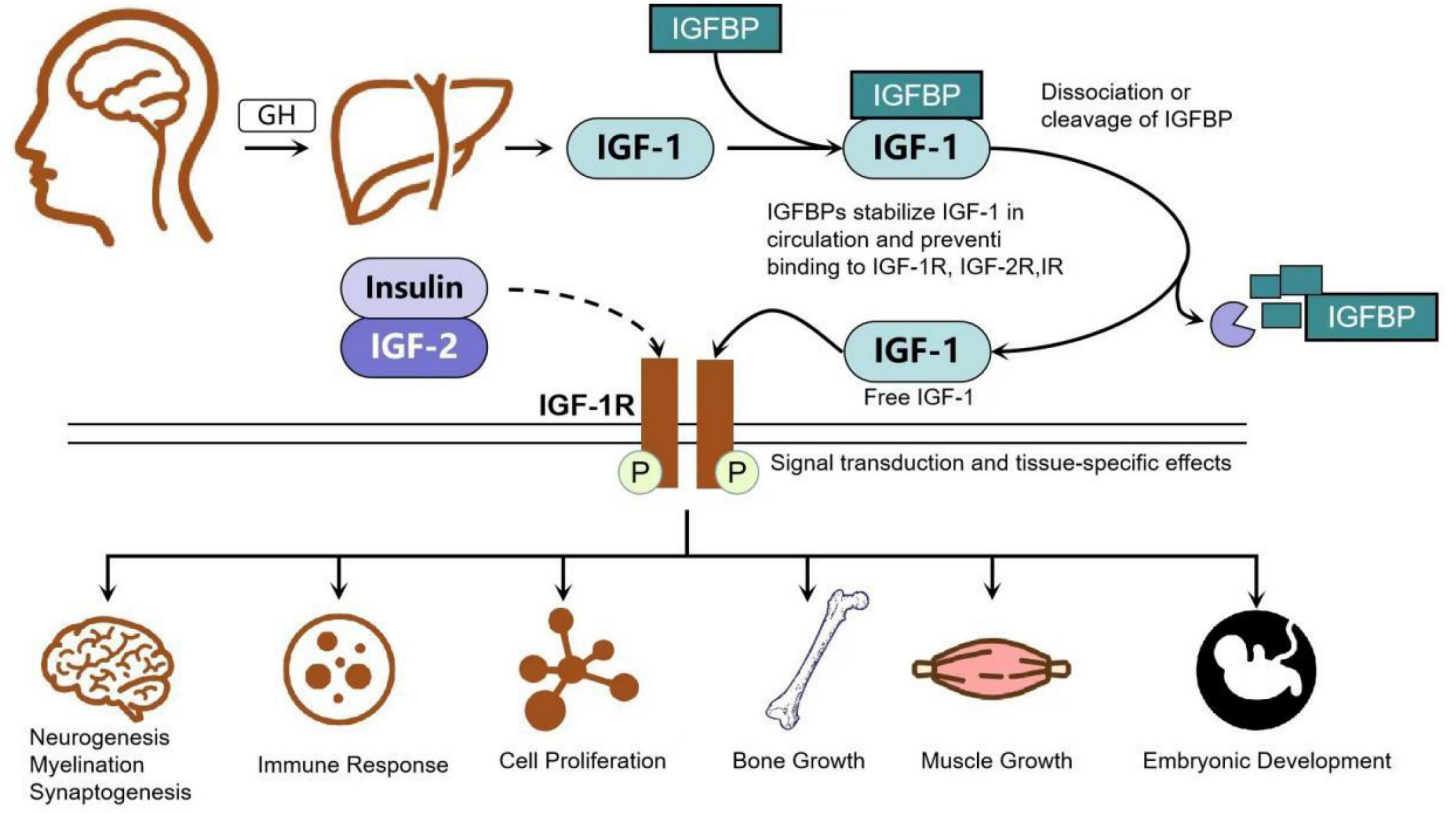
Growth hormone controls the production and release of IGF-1 in hepatocytes, the binding and separation of circulating IGFBP and its selective receptor IGF-1R, and closely regulates IGF-1 signaling.

IGF-1 also regulates oxidative stress and neuroinflammation, two key contributors to ischemic neuronal damage. It attenuates excessive calcium signaling in neural cells (8), promotes activity-dependent functional recovery in chronic stroke (9), and influences systemic cardiovascular outcomes relevant to stroke risk (10). Microglial and astroglial expression of IGF-1 and its binding proteins increases following hypoxia-ischemia, supporting glial survival and inflammatory regulation (11–16). IGF-1 suppresses microglial oxidative stress and inflammatory cytokine release (7, 17–21), while astrocytic IGF-1 and IGFBP-2 expression contributes to oxidative stress resistance (13, 16, 22, 23). Crosstalk with calcineurin, glucose transport pathways, and Na^+^/K^+^-ATPase-related mechanisms further emphasizes its involvement in glial–neuronal interactions and aging-related vulnerability (19, 24–29). Collectively, IGF-1 plays a key role in regulating oxidative stress, inflammation, and brain repair after ischemia (Figure 1), reinforcing its relevance to stroke pathophysiology.

Based on these mechanistic insights, circulating IGF-1 has been proposed as a biomarker associated with ischemic brain injury and recovery (6, 9, 10). However, clinical studies have yielded inconsistent results regarding its association with ischemic stroke incidence or functional outcomes, likely due to differences in study design, patient characteristics, timing of IGF-1 measurement, and adjustment for confounders. These discrepancies underscore the need for a systematic synthesis of available evidence.

Therefore, this systematic review and meta-analysis aimed to (1) assess the association between circulating IGF-1 levels and the risk of ischemic stroke, and (2) examine whether IGF-1 is associated with unfavorable outcomes after ischemic stroke, with particular attention to long-term prognosis.

Unlike previous individual studies, our meta-analysis simultaneously evaluates IGF-1 in relation to both ischemic stroke risk and long-term functional prognosis, with subgroup analyses based on follow-up duration.

## 2 Materials and methods

### 2.1 Data sources and searches

We conducted a systematic search in EMBASE, PubMed, the Cochrane Library and Google Scholar (as a supplementary source) from their inception to June 2021 to identify studies evaluating the effect of IGF-1 on the risk and functional outcomes of ischemic stroke. No language restrictions were applied. The search strategy included the terms “insulin-like growth factor 1,” “IGF-1,” “ischemic stroke,” and “stroke.” Additionally, we reviewed the references of retrieved articles to identify any further relevant studies. In response to reviewer comments, we updated the search in PubMed, Embase, the Cochrane Library, and Google Scholar up to November 2025 using the same search terms and eligibility criteria. The updated search identified several additional reviews, experimental studies, and one cross-sectional clinical study; however, none fulfilled the predefined inclusion criteria (prospective cohort or case-control design assessing ischaemic stroke incidence or post-stroke functional outcome with OR/RR/HR and 95% CI). Therefore, no new primary studies were added to the quantitative synthesis.

### 2.2 Study selection criteria

Published articles were included if they: (1) had a case-control or cohort design, (2) evaluated the association between IGF-1 and ischemic stroke, (3) assessed the impact of IGF-1 on unfavorable outcomes after ischemic stroke, and (4) reported the odds ratio (OR) or risk ratio (RR) with 95% confidence intervals (CI). Duplicate publications or those reporting on the same study population were excluded, with the most recent publication selected. Studies on pediatric populations, articles with insufficient data, and those focused solely on transient ischemic attacks were also excluded.

### 2.3 Literature search

**Figure 2:**
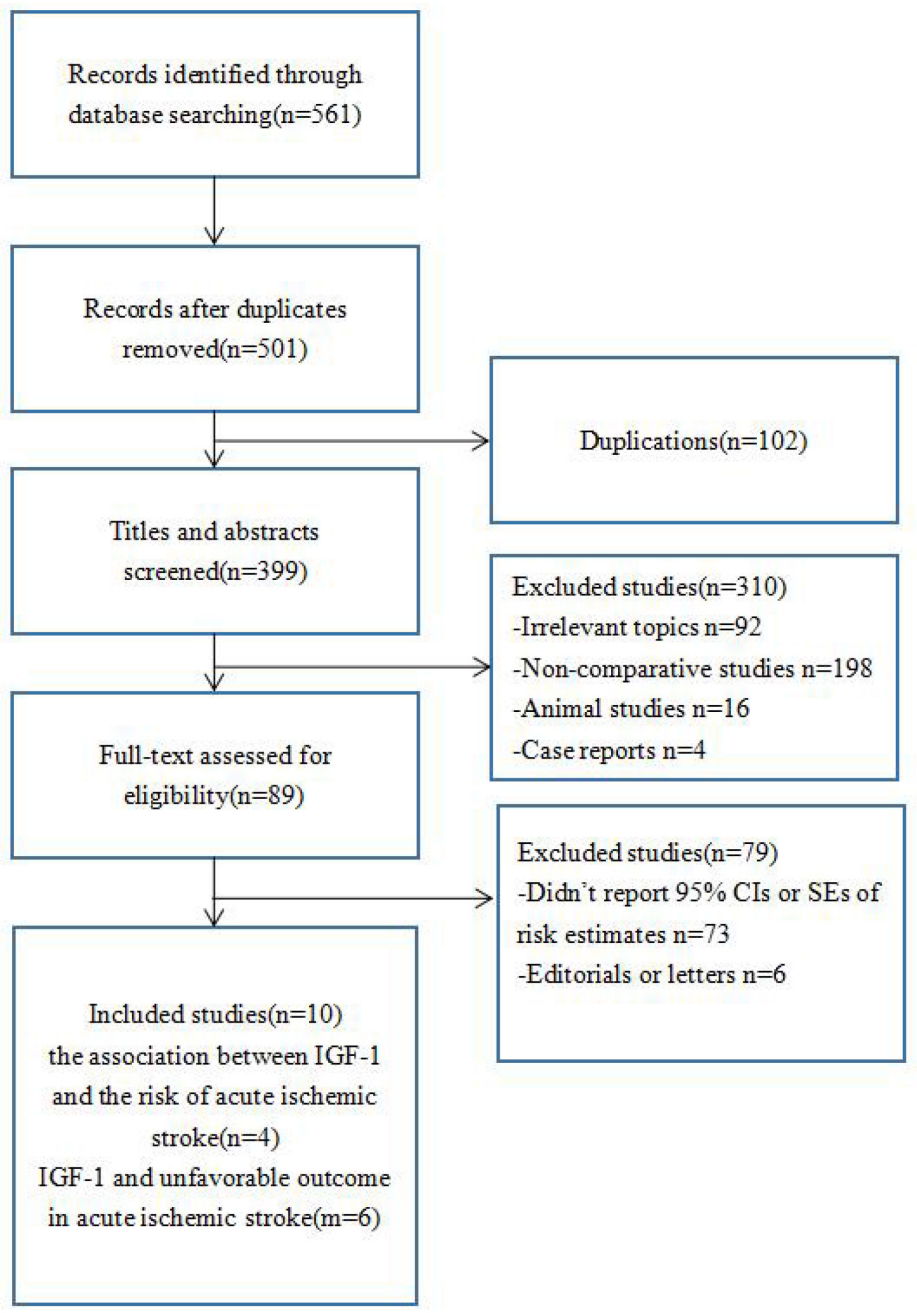
Flow diagram of study selection.

Our systematic literature search identified 10 articles. Four of these studies examined the association between IGF-1 and the risk of acute ischemic stroke, while the other six focused on the relationship between IGF-1 and unfavorable outcomes after acute ischemic stroke. A flow diagram detailing the study selection process is shown in Figure 2. Of the 561 titles identified from the databases, 10 studies were included in the final analysis after reviewing the full text of the remaining studies. The primary reason for exclusion was the lack of 95% confidence intervals (CIs) or standard errors of risk estimates.

### 2.4 Data extraction and quality assessment

Two authors independently assessed the eligibility of all retrieved studies and extracted relevant data using a standardized data form. The data form included the following items: study name (with the first author's name and year of publication), journal name, country, study design, study population, follow-up duration, and adjusted odds ratios (ORs) or risk ratios (RRs) with 95% confidence intervals (CIs). The authors compared their lists, and any disagreements were resolved by consensus. Study quality was assessed independently by two reviewers using the Newcastle-Ottawa Scale (NOS), evaluating participant selection, comparability, and outcome assessment.

### 2.5 Statistical methods

To calculate the summary risk ratio (RR) and 95% confidence interval (CI), we used the most-adjusted RR or odds ratio (OR) and its 95% CI from each study. The overall effect size was recalculated by pooling risk estimates and unfavorable outcomes using the inverse-variance method. Heterogeneity across studies was assessed using *I*^2^ statistics. When substantial heterogeneity was detected, the summary estimate was calculated using a random-effects model. Otherwise, the pooled estimate was derived using a fixed-effects model. Subgroup analyses were conducted based on follow-up duration. All data analyses were performed using STATA 15.1.

## 3 Results

### 3.1 Study characteristics and quality assessment

Descriptive data for the studies included in our analysis are summarized in Tables 1, 2. The studies were conducted in Japan, Belgium, China, the United States, Italy, Korea, and Denmark. Study-specific quality scores are also summarized in Tables 1, 2, with scores ranging from 6 to 10; the median score was 6.

**Table 1:** The association between IGF-1 and unfavorable outcome in acute ischemic stroke (*m* = 6).

**First author, year of publication (reference)**	**Region**	**Result**	**Study population**	**Timing of IGF-1 measurement**	**Timing of outcome measurement**	**OR/RR/HR(95% CI)**	**Rating scale**	**Quality score**
Ann 2011 (68)	Belgium	Low serum IGF-I levels just after ischemic stroke onset are associated with a bad functional outcome.	346 total 255 stroke	6 hours after admission	3 days to 3 months post	HR: 1.7 95% Cl: 1.01–2.86	NIHSS score; modified Rankin scale	5
Tang 2014 (23)	China	Serum IGF-1 levels ≤ 130 ng/mL was as an value indicator for unfavorable functional outcome	268 total 168 stroke	24 hours after admission	Up to 1 year post	OR 3.31 95% CI: 1.87–5.62	NIHSS score; modified Rankin scale	6
Moritz 2017 (26)	America	Low IGF-1 levels (day 8) were independently associated with a decreased risk of an unfavorable outcome	404 stroke	0/8 days after admission	3 months post	Day 8:OR 0.61 95% Cl: 0.37-0.99	modified Rankin scale	6
Licia 2004 (69)	Italy	IGF-1 levels were inversely related to poor outcome (mainly death) at 3 and 6 months.	173 total, 85 stroke	24 hours after admission	Up to 6 months post	OR: 0.8 95% CI: 0.6-1.0	Glasgow Coma Scale and stroke scores	5
Wei 2017 (28)	China	The data showed that low serum IGF-1 levels at admission are associated with a high risk of poor outcome.	120 total, 85 stroke	24 hours after admission	1 year post	OR: 3.35 95% CI: 1.88-6.79	NIHSS score; modified Rankin scale	7
Jeeun 2021 (29)	Korea	A higher serum IGF-1 level is associated with a lower NIHSS score at admission but not at 3 months.	379 stroke	24 hours after admission	3 months post	OR: 0.62 95% CI: 0.27–1.43	NIHSS score	7

**Table 2:** The association between IGF-1 and the risk of acute ischemic stroke (*n* = 4).

**First author, year of publication (reference)**	**Region**	**Design**	**Study population**	**Timing of outcome measurement**	**OR/HR(95% CI)**	**Quality score**
Xiang 2014 (29)	China	Case-control study	221 cases and 200 control subjects;	Time of Admission	OR: 2.16 95% CI: 1.33–3.52	6
Hamidreza 2017 (70)	America	Framingham Study	757 individuals first quintile:151 fifth quintile:150	Across 10 years	HR: 2.3 95% Cl: 1.09–5.06	5
Johnsen 2005 (71)	Denmark	Case-control study	254 cases and 254 control subjects;	Across 3.1 years	OR: 2.06 95% CI: 1.05–4.03	7
Robert 2007 (72)	America	Cohort	370 Ischemic stroke 1,122 Random subcohort	Across 5.6 years	HR: 0.99 95% CI: 0.87–1.12	6

### 3.2 Overall analyses

**Figure 3:**
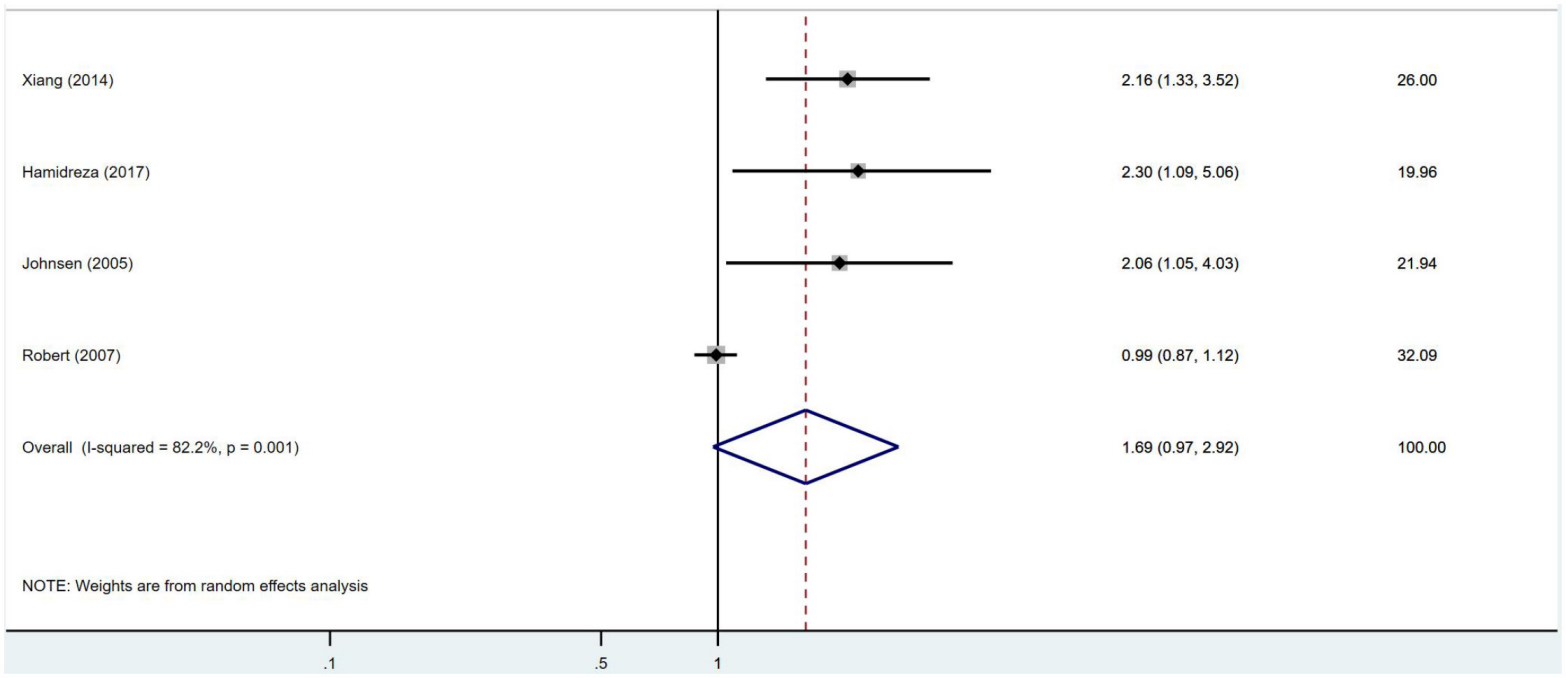
Forest plots shows there was no significant association between IGF-1 and ischemic stroke.

**Figure 4:**
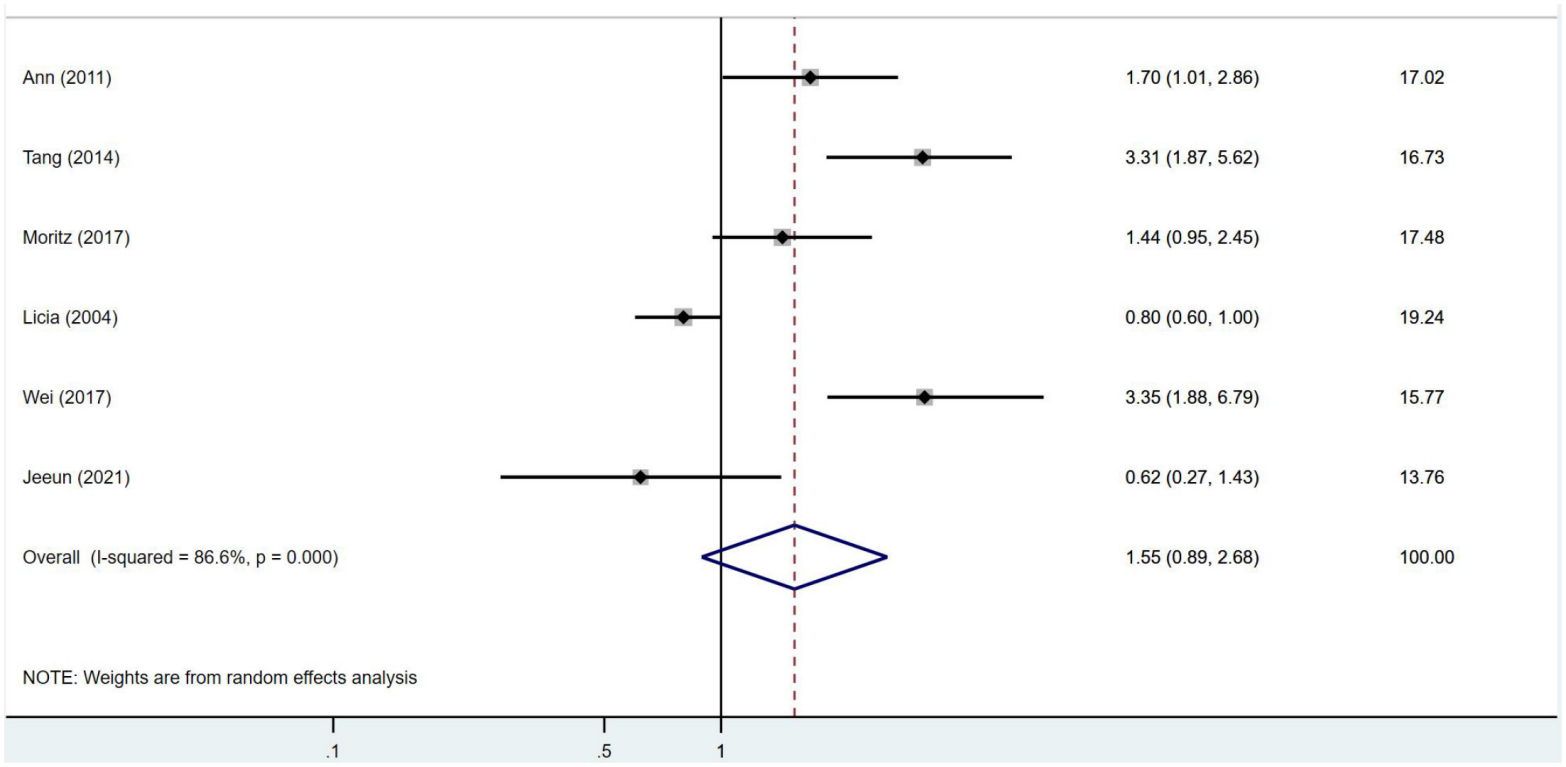
No impact of IGF-1 on unfavorable outcome after ischemic stroke was found.

No significant association was found between IGF-1 and ischemic stroke (RR = 1.69, 95% CI = 0.97–2.92, *I*^2^ = 82.2%, random-effects model, Figure 3), nor was there an impact of IGF-1 on unfavorable outcomes after ischemic stroke (RR = 1.55, 95% CI = 0.89–2.68, *I*^2^ = 86.6%, random-effects model, Figure 4).

**Figure 5:**
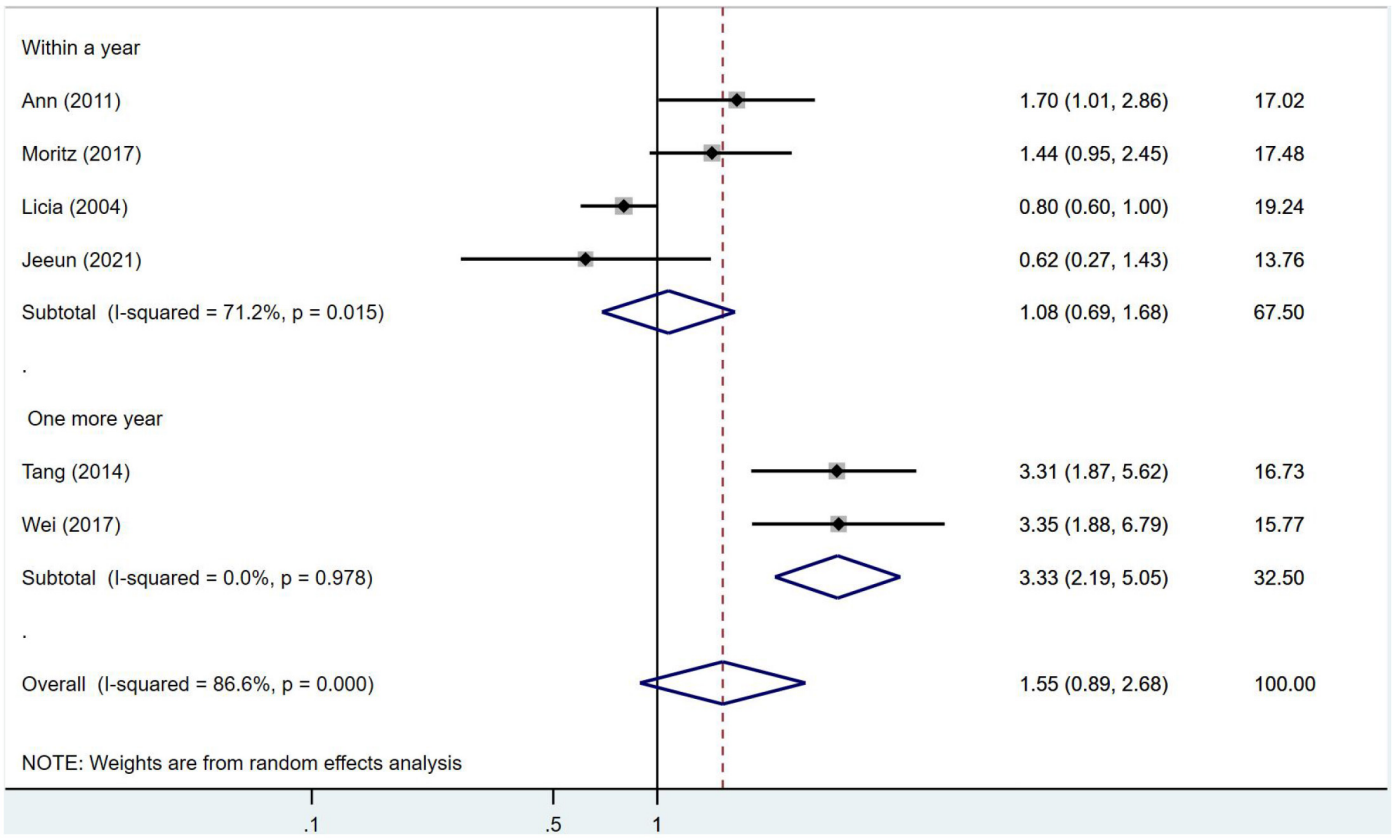
In the subgroup analysis of the effect of IGF-1 on unfavorable outcome after ischemic stroke, IGF-1 was significantly associated with a poor prognosis at more than 1 year after stroke onset.

Follow-up duration affects the severity of post-stroke disability and may better reflect recovery vs. degeneration, therefore subgroup analyses were stratified by 1-year follow-up. However, in the subgroup analysis, IGF-1 was significantly associated with poor prognosis more than 1 year after stroke onset (RR = 3.33, 95% CI = 2.19–5.05, *I*^2^ = 0%, random-effects model, Figure 5). As only a few studies contributed to this subgroup analysis, this finding should be considered exploratory and requires confirmation in future research.

### 3.3 Publication bias

To assess the potential impact of publication bias on the meta-analysis results, we used a funnel plot. The funnel plot of the included studies showed clear left-right symmetry, indicating no evidence of publication bias.

## 4 Discussion

In this systematic review and meta-analysis, we found no significant association between circulating IGF-1 levels and either the risk of acute ischemic stroke or unfavorable short-term outcomes following stroke onset. However, patients with higher IGF-1 levels exhibited an increased risk of poor prognosis at >1 year post-stroke, suggesting potential relevance for long-term outcomes. Given the substantial global burden of stroke-related disability, these findings highlight IGF-1 as a possible biomarker for risk stratification during recovery phases.

IGF-1 plays a fundamental role in growth regulation, metabolism, and tissue repair (30–34). Beyond developmental functions, circulating IGF-1 levels correlate with musculoskeletal performance (35) and show altered regulation across various chronic diseases including cancer and metabolic disorders (36–39). Epidemiological studies have reported inconsistent associations between IGF-1 and stroke risk (40, 41), while preclinical evidence supports its vascular and neuroprotective properties (42, 43). Low IGF-1 levels have been linked to severe neurological injury in acute stroke settings (44), cognitive decline in the elderly (45), and improved recovery with supplementation in animal models (42).

**Figure 6:**
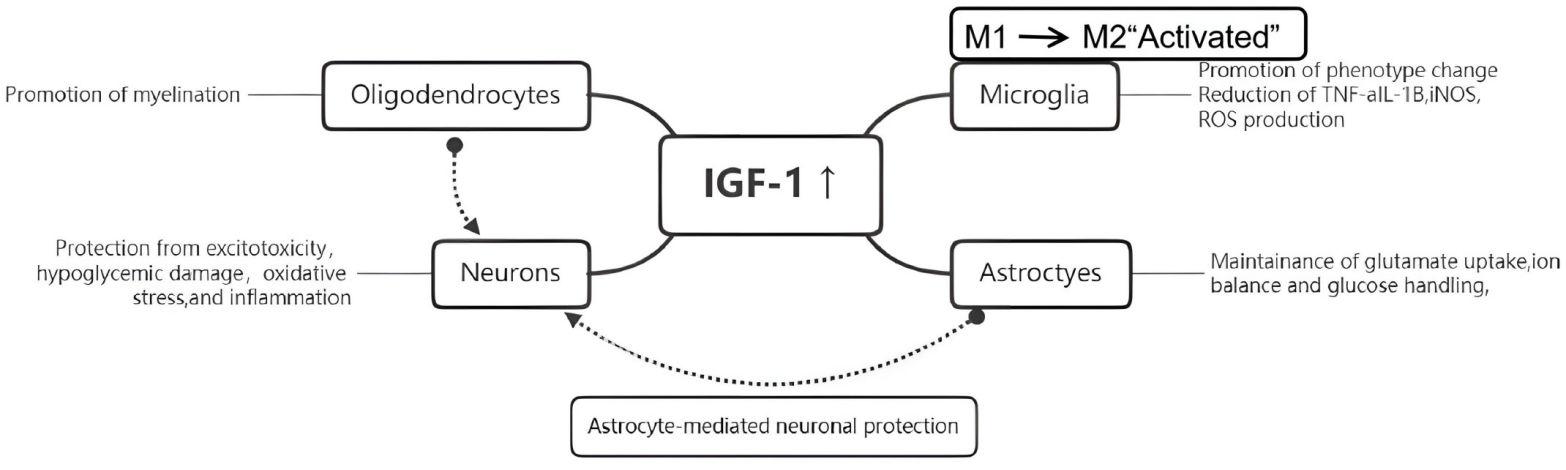
Key cellular functions in the brain are known to be regulated by IGF-1.

Mechanistically, IGF-1 exerts neuroprotective functions through several complementary pathways (Figure 6). First, IGF-1 reduces oxidative stress and inflammation by modulating microglial activation and cytokine production (42, 43). It has been shown to regulate early atherosclerotic processes and endothelial integrity (46). In clinical studies, lower IGF-1 levels have been associated with poorer outcomes after ischemic stroke (47–49), although findings vary according to baseline patient factors (50). Second, IGF-1 binds to its receptor (IGF-1R) and activates downstream PI3K/Akt and MAPK survival pathways (51–55), which enhance neuronal survival, synaptic plasticity, and metabolic support. IGF-1R is widely expressed in neuronal and glial tissues (56–58), and delays in IGF-1 signaling reduce neuroprotective and anti-apoptotic responses (59, 60). Third, IGF-1 promotes angiogenesis and neurogenesis, both essential for long-term neurological restoration after stroke (61–67).

These biological mechanisms provide a rational explanation for our finding: IGF-1 may be more closely associated with long-term neurological remodeling rather than immediate post-stroke injury, consistent with the >1-year subgroup result. Early outcomes are primarily determined by infarct size and reperfusion damage, whereas long-term prognosis reflects ongoing neuronal recovery, synaptic reorganization, and vascular remodeling—processes in which IGF-1 plays major roles.

However, substantial clinical and methodological heterogeneity must be acknowledged. IGF-1 levels are influenced by age, metabolic syndrome, nutritional intake, IGFBP-mediated bioavailability, and hormonal status (36–38, 50), while laboratory assays and sampling times varied widely among included studies. Substantial heterogeneity across studies may reflect differences in study design, population characteristics, timing of IGF-1 measurement, laboratory assays, and inconsistent adjustments for confounding factors such as age, metabolic disorders, and circulating IGFBPs. Therefore, the pooled estimates should be interpreted cautiously. Furthermore, IGF-1 may reflect systemic anabolic capacity rather than disease-specific neuroprotection, complicating causal interpretation.

Our results also showed high statistical heterogeneity. This likely arises from regional diversity, inconsistent confounder adjustments, and variations in timing of outcome assessment. Although subgroup analysis reduced heterogeneity and revealed a statistically significant association, this finding is based on limited data and should be considered exploratory.

Clinically, IGF-1 measurement is feasible and inexpensive, indicating potential as a prognostic indicator for post-stroke management. Patients with persistently low or elevated IGF-1 levels may benefit from closer monitoring and tailored rehabilitation strategies. Nonetheless, practical challenges remain, including assay standardization, reference ranges by age and sex, and integration with existing prognostic models.

This study has several limitations. All included studies were observational; thus, causality cannot be inferred. Residual confounding may persist despite adjusted analyses. Individual patient-level data were unavailable, preventing stratification by stroke subtype, therapeutic interventions, and comorbidity burden. Further well-designed longitudinal cohort studies and interventional trials are required to validate whether modifying IGF-1 signaling could influence long-term stroke prognosis.

Taken together, although preliminary evidence suggests IGF-1 may contribute to long-term neurological outcomes through anti-inflammatory and pro-repair processes, the current findings should be interpreted cautiously. IGF-1 remains a promising prognostic biomarker, but its role as a therapeutic target for ischemic stroke recovery has yet to be confirmed.

## 5 Conclusions

This meta-analysis suggests that oxidative stress-related IGF-1 may be associated with long-term unfavorable outcomes in patients with ischemic stroke, especially beyond 1 year after symptom onset. However, no significant associations were identified between IGF-1 levels and the risk of incident ischemic stroke or short-term functional outcomes.

Given the limited number of studies included, substantial heterogeneity, and the observational nature of the evidence, these findings should be interpreted with caution. IGF-1 may serve as a potential prognostic biomarker for long-term functional decline after stroke, but causality and its therapeutic implications remain uncertain.

Future prospective, large-scale studies with standardized IGF-1 measurements and comprehensive adjustment for confounding factors are needed to confirm the prognostic utility of IGF-1 and clarify its biological relevance in post-stroke recovery.

## Data Availability

The original contributions presented in the study are included in the article/supplementary material, further inquiries can be directed to the corresponding author.
